# Occipito-Cervical Fusion Using Screw Rod Plate System in Craniocervical Pathologies: A Prospective Cohort Analysis of Long-Term Functional and Radiological Outcome With Minimum Two Years of Follow-Up

**DOI:** 10.7759/cureus.100489

**Published:** 2025-12-31

**Authors:** Bharat R Dave, Arjit Vashishtha, Ajay Krishnan, Shivanand C Mayi, Ravi Ranjan Rai, Mirant B Dave, Mukessh Patel, Mahesh Sagar, Mikeson Panthackel, Amritesh Singh, Saurabh S Kulkarni, Yogenkumar Adodariya

**Affiliations:** 1 Spine Surgery, Stavya Spine Hospital and Research Institute, Ahmedabad, IND; 2 Spine, Bhavnagar Institute of Medical Science (BIMS), Bhavnagar, IND; 3 Neurosurgery, Stavya Spine Hospital and Research Institute, Ahmedabad, IND

**Keywords:** basilar invagination, craniovertebral junction, intra-operative 3d ct, navigation, occipito-cervical fusion

## Abstract

Introduction: Occipito-cervical fusion is a standard treatment for craniovertebral junction (CVJ) instability and myelopathy. The technique has evolved from onlay bone grafting with halo immobilisation to rigid internal fixation with pedicle screws and rods construct, allowing early mobilisation and improving fusion rates. We describe our experience with the posterior-only surgical technique using enabling technologies, avoiding facet joint exposure and its associated complications.

Methodology: A total of 19 patients who underwent occipito-cervical fusion for CVJ pathologies were evaluated. These patients were followed up for a minimum of 24 months. Clinical assessment included Visual Analog Scale (VAS), Neck Disability Index (NDI), Nurick grade, and modified Japanese Orthopaedic Association (mJOA) scores. Radiological parameters, McGregor slope, occiput-C2 angle (O-C2), C2-C7 lordosis, and posterior occipito-cervical angle (POCA), were evaluated on lateral radiographs. Fusion was confirmed via CT scan by the presence of bony trabeculae between the occiput and C2 lamina.

Results: Of the 19 patients, 18 (94.7%) achieved radiological fusion on follow-up CT scan, showing bony trabeculae often forming around the rods. Clinically, mean VAS improved from 7.4 to 1.8, NDI from 38.6 to 15.8, Nurick grade from 3.2 to 0.9, and mJOA from 10.6 to 15.4. Radiologically, the McGregor slope, O-C2 angle, and POCA showed significant improvement postoperatively, indicating better alignment.

Conclusion: Posterior-only occipito-cervical fusion using modern technologies results in high fusion rates, clinical improvement, and radiological correction, while avoiding the morbidity associated with anterior approaches and facet joint exposure.

## Introduction

Occipito-cervical fusion, as its name suggests, is the process of fixation of the cranio-vertebral junction (CVJ) with bone grafting to achieve bony fusion between the occiput and the axis or the sub-axial cervical spine. The procedure is widely employed as an umbrella treatment for various CVJ pathologies of congenital, inflammatory, traumatic, infective, or degenerative aetiologies. Foerster described occipito-cervical fusion using a fibula strut graft in 1927 [1]. The technique has evolved immensely over the years from on-lay bone grafting with or without wires, rods, or pins [2-4], providing unstable fixation that demands postoperative immobilization, despite which failure rates have been reported to be high [5,6]. The advent of screws, plates, and rod constructs [7-10], which provide rigid fixation, obviates the need for prolonged immobilization and results in much better fusion rates. This evolution has improved the patient outcomes with reduced complications.

In addition to instrumentation, various methods for achieving occipito-cervical bone grafting have been described. Autologous iliac bone graft has been described to be placed over a trough made in the base of the occiput and fixed with wires or cables [11]. Paired autologous ribs have also been described to be used as a structural bone graft in place of the iliac crest, with the advantage of fitting closely to the anatomy of the occipito-cervical junction [12]. For the reduction of basilar invagination and irreducible atlanto-axial dislocation with atlas assimilation, a novel technique called DCER (Distraction, Compression, Extension, and Reduction) has been described, where the joint between the occipital condyles and the superior articular surface of the atlas lateral mass is explored. A spacer is placed after denuding the cartilaginous surface, which acts as a fulcrum, over which the reduction of the basilar invagination or the atlanto-axial dislocation takes place [13]. However, access to the facet joint is difficult and is associated with significant blood loss, increased surgical time, and chances of inadvertent vertebral injury.

We present our institutional experience of 19 cases of CVJ pathologies, including basilar invagination and atlanto-axial dislocation, who presented with symptoms of myelopathy of variable duration, with or without supra-axial neck pain, managed with occipito-cervical fusion using intra-operative three-dimensional (3D) CT scan and navigation. Neck extension and posterior distraction were performed under intra-operative neuromonitoring (IONM), while connecting the rods from the occiput to screws in the subaxial cervical spine in all cases to reduce basilar invagination and retro-odontoid tilt. This manoeuvre relieves the compression over the spinal cord at the CVJ. A copious amount of autologous cancellous bone graft was harvested from the posterior superior iliac spine, which was placed over the decorticated occiput and laminae to achieve fusion. These patients were evaluated clinically and radiologically for a minimum follow-up period of 24 months. We believe that with the optimal use of enabling technologies, such a complex surgery can be performed efficiently and safely, obviating the need for facet exploration and associated complications.

## Materials and methods

This was a Prospective Cohort Analysis at the Stavya Spine Hospital and Research Institute, Ahmedabad, Gujarat, India. The study was approved by the Institutional Ethics Committee of Stavya Spine Hospital and Research Institute (study protocol code: SSHRI/CS/NS/RetroOCF/BRDDD/50/07.22) and is registered with the Clinical Trials Registry - India (registration number: CTRI/2022/09/045401).

Study population

The primary inclusion criteria were craniovertebral pathologies (basilar invagination or atlanto-axial dislocation) warranting occipito-cervical fusion surgery, only posterior surgeries with screws, plates, and rods, and patients willing to undergo 24-month follow-up. Patients were excluded if they were lost to follow-up, underwent anterior surgery or revision surgery, if sublaminar wires were used instead of screws, plates, and rods, or if there was a presence of infection.

A total of 32 patients undergoing occipito-cervical fusion at our institution between July 2022 and September 2023 were enrolled. Of these 32 patients, we were able to follow up 19 patients for at least two years, till September 2025. 

Study procedure

**Figure 1:**
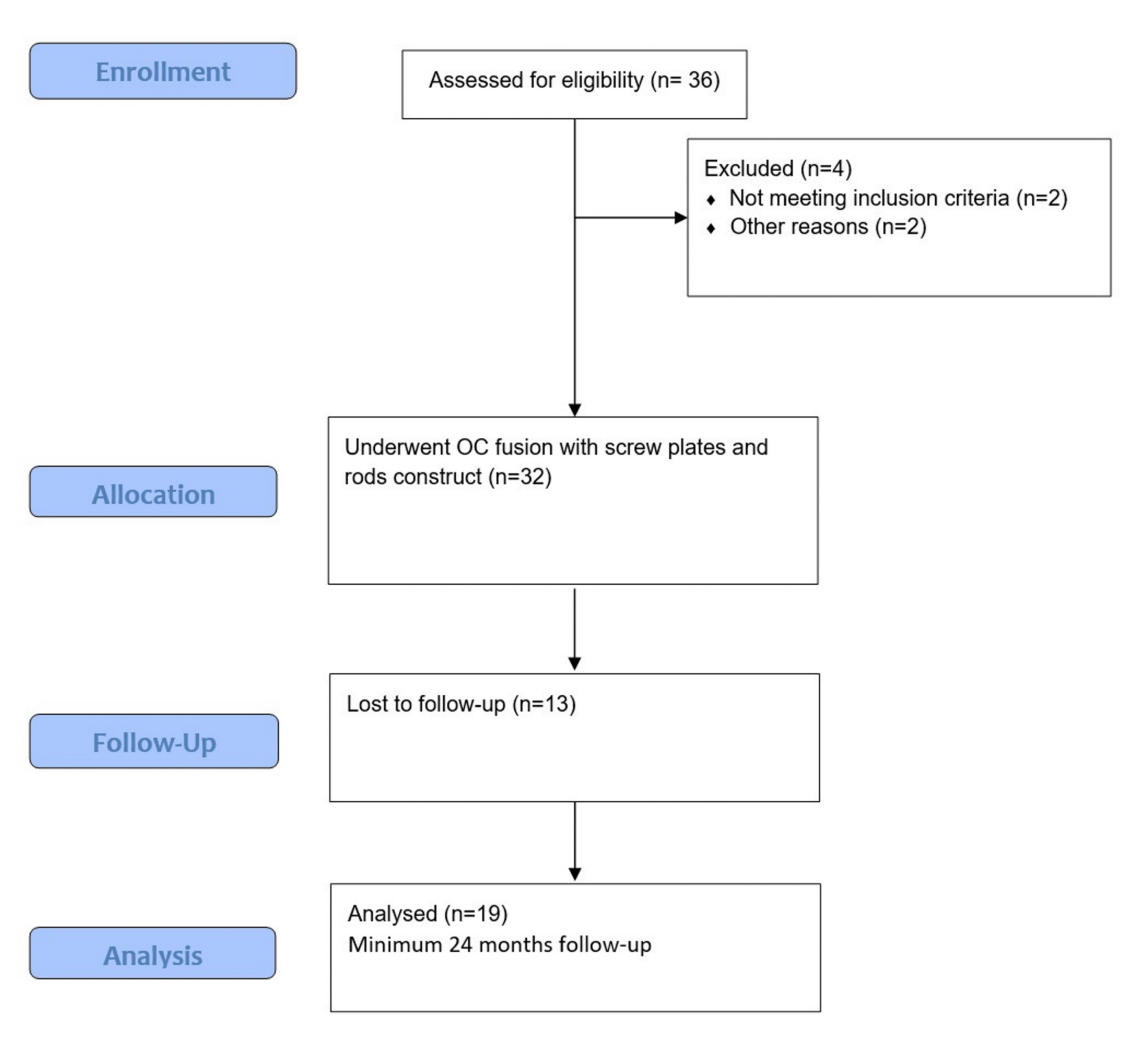
A participant flow chart diagram summarising the enrollment, follow-up and analysis. OC: occipito-cervical

Adhering to our institutional protocol, these patients were regularly followed up at six weeks, six months, 12 months, and 24 months postoperatively. The participant flow chart diagram in Figure 1 summarises the enrollment, follow-up, and analysis. Clinically, pain intensity and disability were evaluated using the Visual Analogue Scale (VAS) [14] and the Neck Disability Index (NDI) [15]. Nurick grades [16] and modified Japanese Orthopaedics Association (mJOA) [17] scores were noted to represent the severity of myelopathy at the time of presentation and at postoperative follow-up visits. VAS is for the objective assessment of pain, while NDI reflects the functional impairment due to the pathology. Similarly, Nurick grades and mJOA score represent how much functional impairment is present due to the myelopathy. While VAS, NDI, and Nurick grade were available for open access, the mJOA score has been used with permission from its author [17]. 

Radiological parameters like McGregor slope, occiput-axis lordosis (O-C2 angle), subaxial cervical lordosis (C2-C7 lordosis), and posterior occiput-cervical angle (POCA) were calculated on lateral radiographs. The status of fusion was assessed by CT scan at the postoperative 24-month follow-up visit. 

Surgical technique 

**Figure 2:**
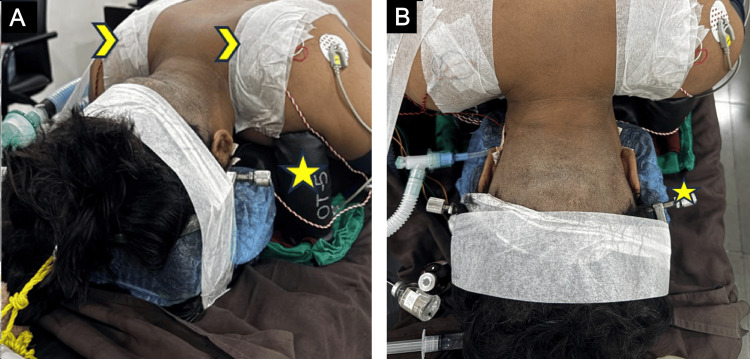
Clinical photograph after the patient is positioned prone on the radiolucent operating table, facilitating intra-operative imaging. Reverse Trendelenburg position under IONM. (A) A shoulder bolster (yellow star) was used to support both shoulders and prevent them from hanging down, keeping them out of the field of view of the cervical spine for better intra-operative imaging. Chevron arrows point towards the micropore strapping of the shoulders to pull them caudally. (B) Shaving of the occipital area is done, and the head is stabilised with Graden-Wells tong's traction (yellow star). IONM: intra-operative neuromonitoring

All the patients planned for occipito-cervical fusion were counselled regarding the severity of the pathology and the need for surgical intervention. Informed consent was taken after explaining the vascular and neurological injury risk as well as the chances of needing post-operative ventilator support. The patients were intubated with extra care to avoid excessive neck manipulation. A video laryngoscope was used for cases with difficult intubation. A Gardner-Wells tong was applied intraoperatively to stabilise the head and neck. Intraoperative neuromonitoring was used in all the cases. Motor evoked potentials (MEPs) were checked in the supine position; the patient was then placed in the prone position. Gentle traction with a 2 kg weight was applied using tongs to stabilise the head. The neck was placed in a neutral position. The reverse Trendelenburg position was given to reduce venous bleeding (Figure 2). 

**Figure 3:**
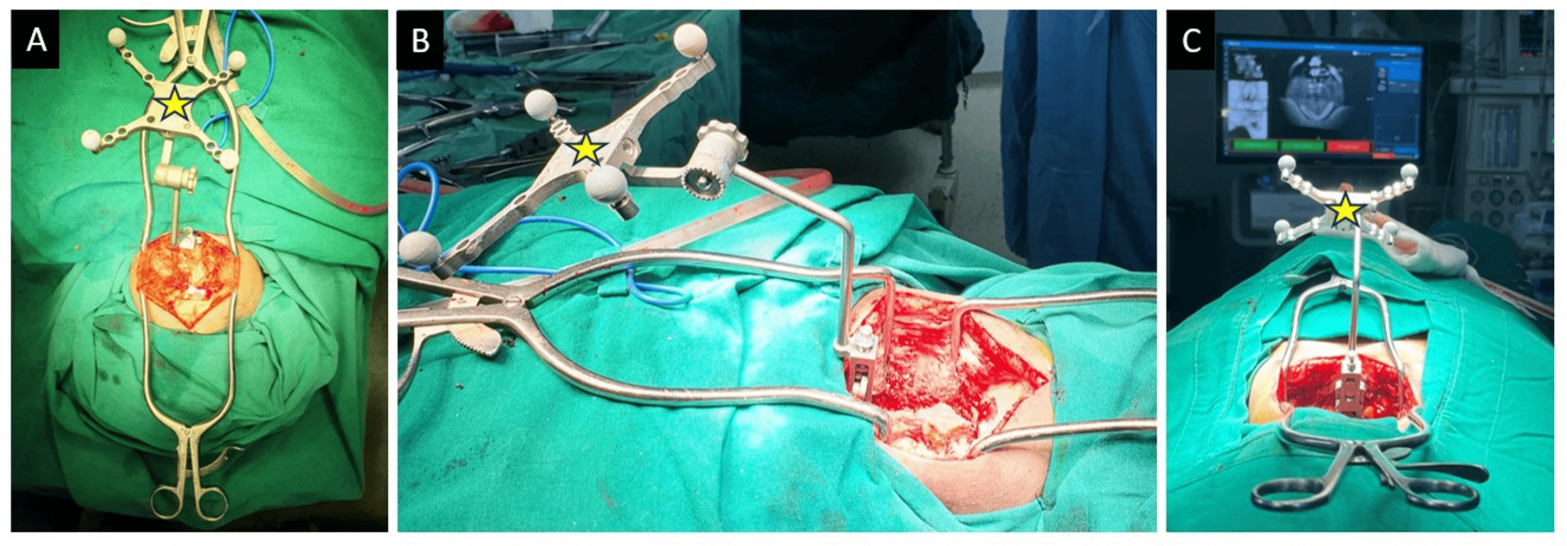
Navigation frame attached with the help of a clamp to the spinous process.

**Figure 4:**
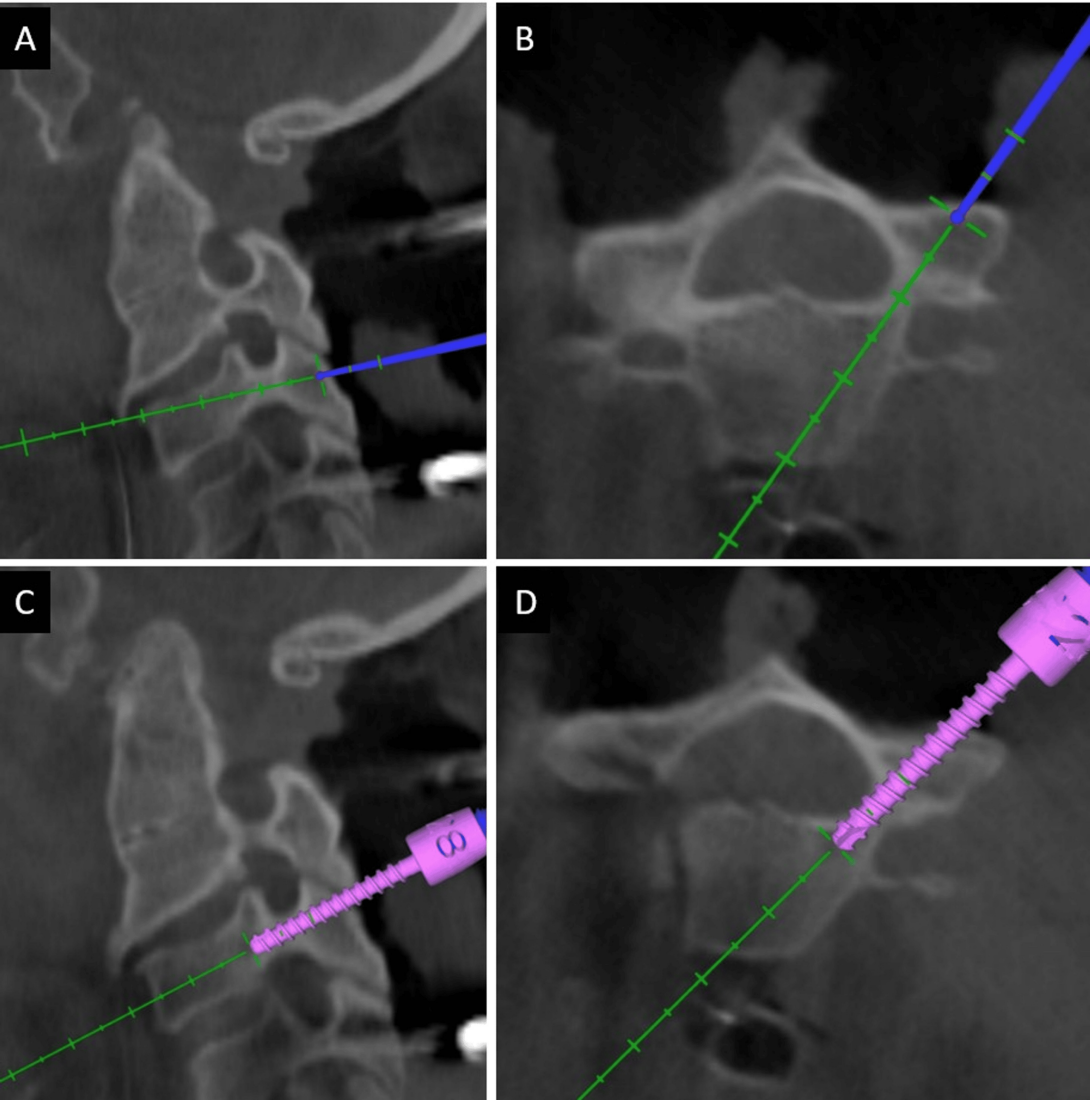
Three-dimensional navigation guiding the best possible trajectory for C4 pedicle screw in (A) sagittal and (B) axial plane. Placement of the C4 pedicle screw through the planned trajectory with real-time localization in (C) sagittal and (D) axial plane.

**Figure 5:**
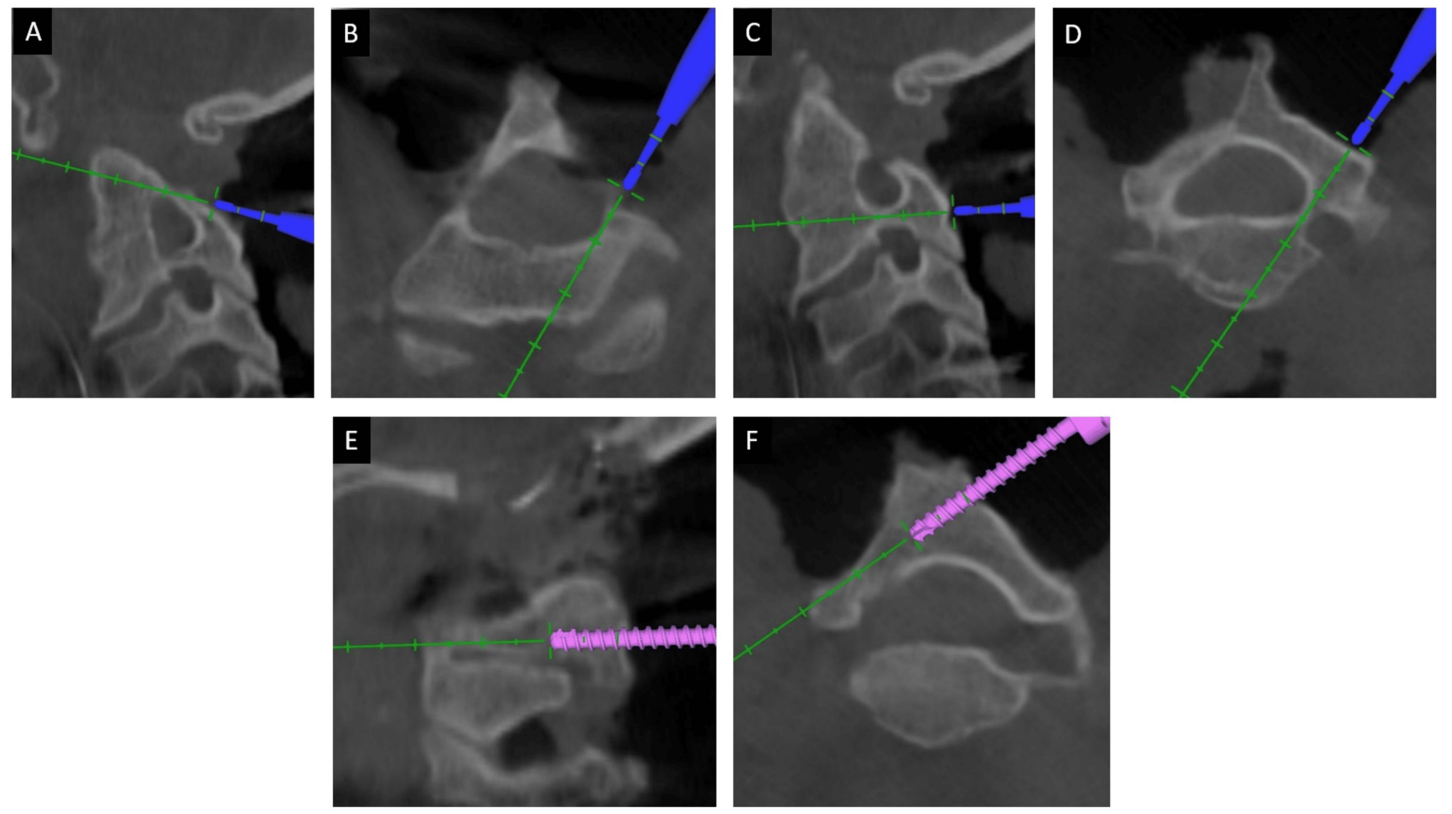
A case where C2 and C3 pedicles were very narrow and it was not possible to place a pedicle screw in either one of them. (A) Sagittal and (B) axial images at the level of C2 pedicle and (C) sagittal and (D) axial images at the level of C3 pedicle. Hence, it was decided to place the C2 laminar screw, shown in images (E) in the sagittal plane and (F) in the axial plane.

Posterior midline exposure of the CNJ was done with subperiosteal dissection of the occiput and subaxial cervical spine. Foramen magnum decompression was done in all of the cases using an ultrasonic bone scalpel. Approximately 1cm of occipital bone near the foramen magnum was cut with a bone scalpel and removed. Approximately 1.5 cm of the posterior arch of the atlas on either side was also removed. The navigation frame was attached with a multi-anchor clamp holding onto the spinous processes of the subaxial cervical spine (Figure 3); a 3D CT scan spin was taken from occiput to subaxial cervical spine. Using navigated tools, pedicle screws were placed bilaterally in C2 whenever possible and at least one more level in the subaxial cervical spine (Figure 4). Initially, all the screws were partially inserted into the pedicles. In cases where C2 pedicle screw placement was not possible, an interlaminar screw was placed into the broad C2 lamina (Figure 5). A second 3D CT scan was performed to assess the position and trajectories of the partially inserted screws. If a medio-lateral or cranio-caudal breach was found in any of the screws, it was removed, and a new pedicle screw was placed at a different trajectory at the same level or at another level. 

Occipital plate/rods were contoured to provide extension and fixed to the cervical anchors bilaterally. With the help of anaesthetists, gentle neck extension was given, and occipital plate/rods were fixed to the occiput with 8-12mm cortical screws. MEPs were checked after this manoeuvre. If the signals were found to be okay, the screw nuts at the cervical spine were loosened, and gradual distraction was done between the occiput and the cervical spine. MEPs were rechecked to confirm that they were the same as before, and the final tightening was done.

**Figure 6:**
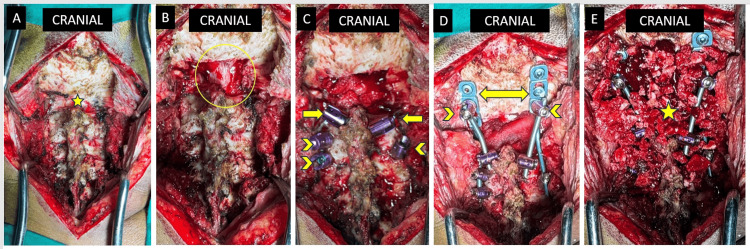
Sequential intra-operative images taken during the procedure. (A) Subperiosteal exposure of the occiput and cervical spine. The craniovertebral junction (yellow star) is carefully dissected, maintaining haemostasis. (B) Brainstem and the upper part of the spinal cord (yellow circle) are seen following foramen magnum decompression. (C) Image taken following instrumentation of the cervical spine. Arrows point towards the C2 laminar screw on either side, while chevron arrows point towards C3 and C4 pedicle screws. (D) Occipito-cervical construct with occipital plate/rod (arrows) fixed to occiput along with a polyaxial pedicle screw on either side (chevron arrow) placed through the occiput plate hole. A four-rod construct is made by connecting the occipital plate/rod to the C3 and C4 pedicle screws and the occiput pedicle screws to the C2 laminar screws. (E) Cortico-cancellous autologous iliac crest bone graft was placed over the construct after decortication.

The occiput and the cervical spine laminae till the last instrumented level were decorticated with a burr to prepare the fusion bed. Autologous cancellous bone graft was harvested from the posterior superior iliac spine. An adequate amount of bone graft was packed between the occiput and the cervical spine between the two rods. A layer of Gelfoam was placed over the graft. Wound closure is done in layers with a negative suction drain. Intra-operative images at various steps during the procedure are visualised in Figure 6.

The complete surgical procedure was performed under intraoperative neuromonitoring, with continuous somatosensory evoked potential (SSEP) and MEP monitoring during the critical steps, especially during the reduction manoeuvre. The steps of the reduction manoeuvre were reversed if a signal drop was noticed, and the checklist for IONM signal drop was followed. 

Postoperatively, tongs were removed immediately, and patients were mobilised on postoperative day 2 with a hard cervical collar. Regular follow-up of patients was performed at six weeks, six months, 12 months, and 24 months, including clinic-radiological evaluation.

## Results

A total of 19 patients, eight male and 11 female, were followed up and analysed in the study. The age at the time of surgery ranged from 17 to 66 years, with a median of 40 years. Of the 19 patients, all of whom had congenital anomalies such as basilar invagination or irreducible atlanto-axial dislocation, three were associated with Arnold-Chiari malformation (ACM). The patients were followed up for 24 months after surgery. Fusion was observed in 18 patients, characterized by bony trabeculae formation, as evident on CT scans. Although the amount of bony trabeculae formation varied among cases, it was most commonly seen around the connecting rods. One patient presented with implant failure in the form of a broken connecting rod on the left side, with mild suboccipital pain at 10 months follow-up, who eventually underwent revision surgery. The basic details of all patients are summarised in Table 1.

**Table 1:** Summary of all the patients included in the study F: female; M: male; ACM: Arnold Chiari malformation; BI: basilar invagination; AAD: atlantoaxial dislocation; O-C2: occiput to C2; O-C3: occiput to C3; O-C4: occiput to C4; O-C5: occiput to C5

S. No.	Age	Sex	Diagnosis	Associated conditions	Fusion levels	Complications	Follow-up
1	47	F	BI	ACM	O-C4		24 months
2	43	F	BI	AAD	O-C3		24 months
3	40	F	BI	AAD	O-C4		26 months
4	62	F	AAD		O-C4	Transient Dysphagia	24 months
5	36	M	AAD		O-C5		28 months
6	54	F	AAD		O-C5	Transient Dysphagia	24 months
7	24	M	BI	CSM	O-C4		27 months
8	66	M	AAD		O-C5	Transient Dysphagia	24 months
9	19	M	BI		O-C2		26 months
10	48	F	BI		O-C5	Transient Dysphagia	24 months
11	17	M	BI	AAD	O-C3		24 months
12	38	F	BI	ACM	O-C4		24 months
13	23	M	BI		O-C3	Implant failure	10 months
14	40	M	BI	AAD	O-C4		24 months
15	42	F	AAD		O-C2		24 months
16	28	F	BI		O-C4		24 months
17	36	M	BI	ACM	O-C4		28 months
18	47	M	AAD		O-C3	Transient Dysphagia	24 months
19	38	F	BI		O-C4		26 months

Thirteen patients had radiological evidence of basilar invagination (BI), of whom four were associated with atlanto-axial dislocation (AAD), three had concomitant syrinx formation in the spinal cord, and one had associated cervical spondylotic myelopathy (CSM). The remaining six patients had irreducible atlanto-axial dislocations. 

Clinically, all patients showed improvement in pain and myelopathy. The mean VAS and NDI scores were 7.4±1.2 and 38.6±7.4 preoperatively and 1.8±1.1 and 15.8±3.7 at follow-up. All patients had significant functional disability due to myelopathy, with mean mJOA and Nurick Grades of 10.6±2.3 and 3.2±0.7, respectively, at presentation, which improved to 15.4±2.7 and 0.9±1.0, respectively, at follow-up. 

Radiologically, the mean McGregor slope was 18.6°±10.2° pre-operatively and 2.96°±9.5° at follow-up. The occiput to axis lordosis (O-C2 angle) was measured to have a mean value of -12.6°±10.8° (negative value indicating kyphosis) preoperatively and 12.3°±32.7° at follow-up radiographs. Similarly, the subaxial cervical lordosis (C2-C7 angle) had a mean value of 30.2°±8.5° preoperatively and 15.4°±26.5° at follow-up radiographs. The preoperative mean POCA was 59.7°±16.9°, and the mean value at follow-up was 101.1°±27.1°.

**Figure 7:**
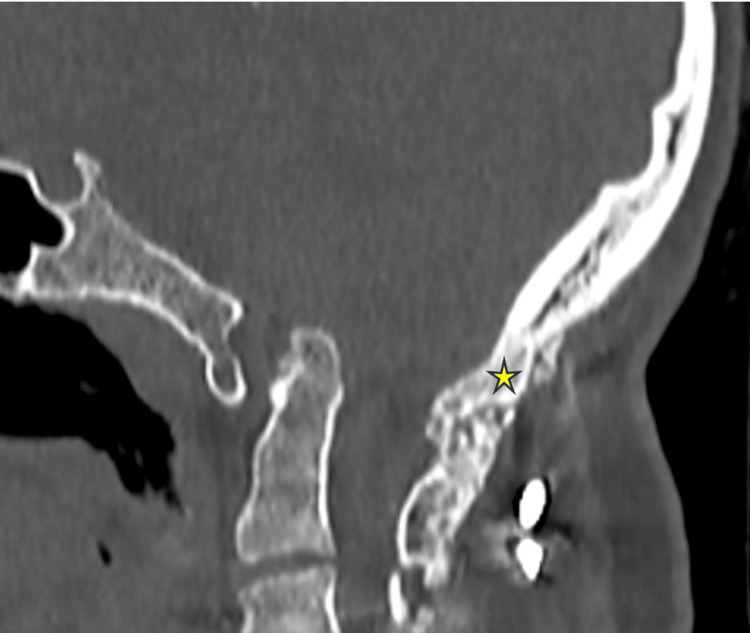
A follow-up CT scan image of cranio-vertebral junction with the star mark showing solid fusion mass with thick trabeculae formation from occiput to C2 lamina.

On follow-up CT scans of the CVJ in these patients, 18 out of 19 patients exhibited fusion characterized by the formation of bony trabeculae between the occiput and the cervical spine (Figure 7). The fusion rate was calculated to be 94.7%. One patient presented with a broken rod in-situ at 10 months of follow-up. 

Certain minor complications, such as dysphagia, headache, dizziness, nausea, and vomiting, were observed in the immediate post-operative period in some patients, but all were transient and subsided within one week of surgery. No major long-term complications were observed in these cases.

**Figure 8:**
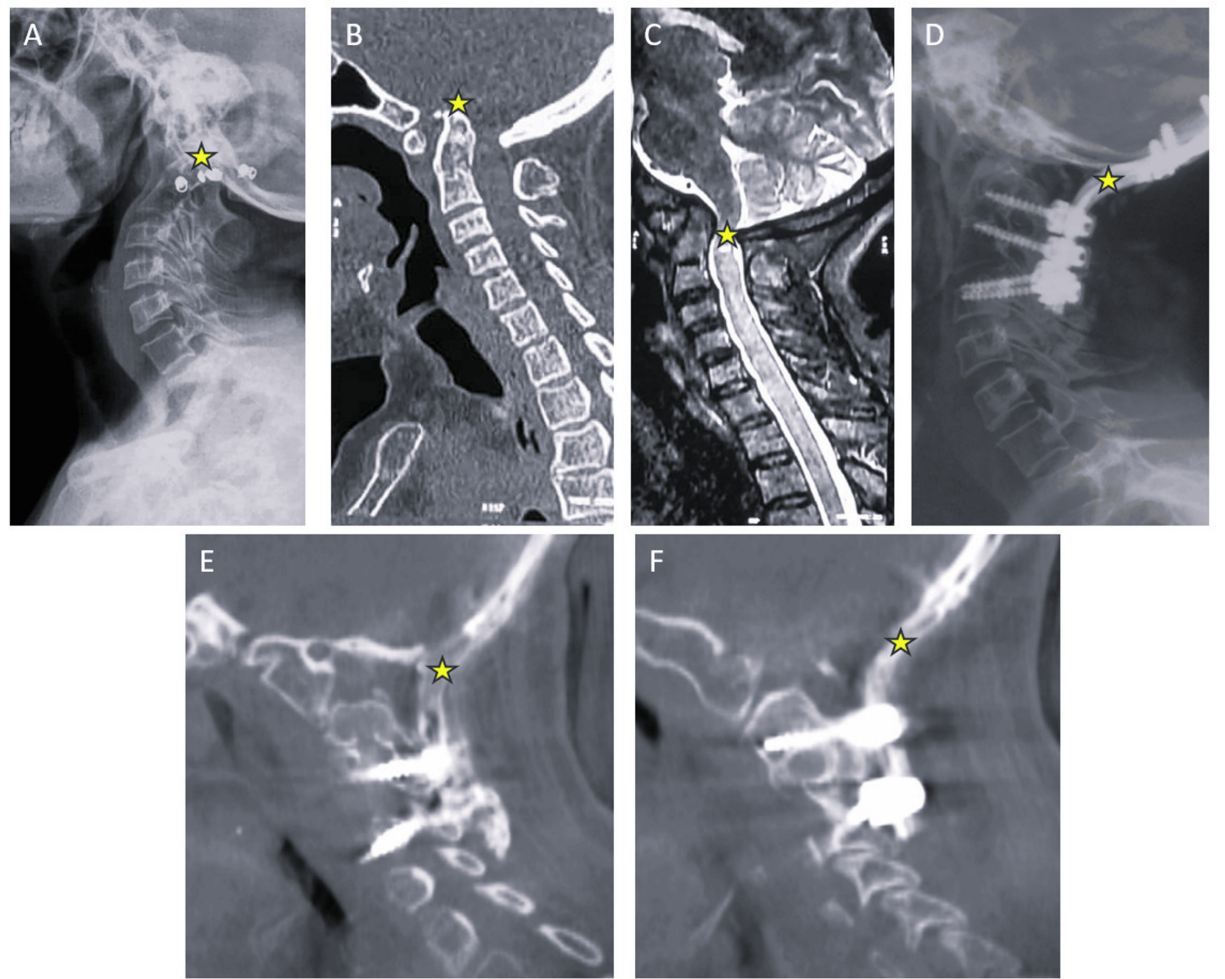
Illustrative case of a patient presented with basilar invagination and irreducible atlanto-axial dislocation (A) Lateral radiographs of the cervical spine with a star mark representing odontoid invagination. (B) Mid-sagittal CT scan showing basilar invagination (yellow star) and an increased atlanto-dens interval. (C) Mid-sagittal MRI image showing myelomalacia (yellow star) at the cranio-vertebral junction. (D) Postoperative lateral radiograph of the patient, after foramen magnum decompression and occipito-cervical fusion. (E) and (F) Parasagittal images from the two-year follow-up CT scan representing the bony trabeculae formation.

Radiograph and CT images of one representative case have been demonstrated in Figure 8, from the time of presentation till the 24-month follow-up. The clinical and radiological parameters at presentation (preoperative) and at the 12-month follow-up are compared in Table 2.

**Table 2:** Mean values of clinical and radiological parameters at presentation (preoperative) and at 12-month follow-up. p-value < 0.05 shows statistical significance VAS: Visual Analog Scale; NDI: Neck Disability Index; mJOA score: modified Japanese Orthopaedic Association score; O-C2 angle: occiput to axis lordosis; C2-C7 lordosis: subaxial cervical spine lordosis; POCA: posterior occiput-cervical angle.

	VAS	NDI	mJOA score	Nurick grade	McGregor slope	O-C2 angle	C2-C7 lordosis	POCA
Preoperative. mean±SD	7.4±1.2	38.6±7.4	10.6±2.3	3.2±0.7	18.6°±10.2°	-12.6°±10.8	30.2°±8.5°	59.7°±16.9°
12-month follow-up. mean±SD	1.8±1.1	15.8±3.7	15.4±2.7	0.9 ±1.0	2.96°±9.5°	12.3°±32.7°	15.4°±26.5°	101.1°±27.1°
p-value	p < 0.0001	p < 0.0001	p < 0.0001	p < 0.0001	p < 0.0001	p = 0.0033	p = 0.0262	p < 0.0001

## Discussion

CVJ pathologies can cause either instability or myelopathic features. Occipito-cervical fusion is a time-tested solution for complex pathologies such as basilar invagination and irreducible atlanto-axial dislocation. Multiple occipito-cervical instrumentation and fusion techniques have been described over the years. 

Initial attempts at occipito-cervical fusion were made with onlay morselized bone graft, followed by halo immobilisation, with fusion rates ranging from 75% to 89% [2,18]. Brattström and Granholm first reported occipito-cervical fusion for atlanto-axial dislocations in 28 patients with rheumatoid arthritis, using sublaminar wires and bone cement, obviating the need for external immobilisation with a halo and enabling early mobilisation. The mean follow-up period was 15.9 months [19]. Wertheim and Bohlman, in their study of 13 patients with an average follow-up of 3.6 years, advocated the use of structural iliac crest bone graft with sublaminar wires for arthrodesis and early mobilisation [3]. Although the majority of reports employ morselized iliac crest bone graft, some have reported use of structural rib grafts [2,20]. Wang and Wang recommended an improved bone grafting method using autologous iliac crest corticocancellous bone graft, based on their experience with 52 cases with a minimum 12-month follow-up [21]. The advent of plates, rods, and screws enabled rigid internal fixation, providing early mobilisation, better fusion rates (95-100%), and lower failure rates [22-24]. The fusion rate among 19 patients of our study, who were followed for 24 months, was 94.7%, which is comparable to existing literature. 

Winegar et al. reported in their review of 34 articles and 799 patients that rigid fixation was associated with greater patient satisfaction and that successful fusion correlated significantly with neurological improvement [24]. Deutsch et al. found in their retrospective review of 58 patients with a mean follow-up of 36 months that no patient improved by more than 1 Nurick grade, and there was a trend toward patients with lower preoperative Nurick grades being more likely to improve [20]. However, in our study, all patients showed functional recovery, with a mean improvement in Nurick grade of 2.27. 

Many techniques have been described over the years to reduce basilar invagination and atlanto-axial dislocation. Goel et al. described a method for craniovertebral junction realignment by manually distracting the atlanto-axial joint and placing a metal spacer [25]. However, the inconsistent correction of the atlanto-axial dislocation was a limitation. Jian et al. later described intraoperative distraction between the occiput and C2 pedicle screws in cases of basilar invagination with atlas assimilation and atlanto-axial dislocation [26]. Chandra et al. devised the technique of DCER and compared the preoperative and postoperative status of 148 patients with basilar invagination, where a metal spacer was placed to distract the atlanto-axial joint, following which compression and extension were done, keeping the metal spacer as a fulcrum, to correct the vertical translation as well as the retro-odontoid tilt [13]. Recent advances in reduction techniques have obviated the need for odontoidectomy [27]. Our technique involves posterior distraction between the occiput and C1 or C2, along with extension. We believe accurate placement of cervical pedicle screws under navigation guidance can provide firm anchorage, enabling the reduction manoeuvre to be effective. This was followed by preparation of the bone fusion bed and autologous iliac crest cancellous morselized bone grafting. 

The radiological parameters around the CVJ also showed improvement post-operatively. The mean postoperative McGregor slope was 2.96±9.48°. The CVJ was found to be in kyphosis in most patients, with a mean preoperative O-C2 angle of -12.6±10.8°, which improved to a postoperative mean of 12.3±32.7°, suggesting lordosis obtained by an extension manoeuvre before fixation. The subaxial cervical spine was found to be hyperlordotic, with a mean C2-C7 lordosis of 30.2±8.5°, which decreased to a mean postoperative value of 15.5±26.5°. The mean preoperative POCA value of 59.7±16.9° was suggestive of cranial settling, which improved to a mean post-operative value of 101.1±27.1°. 

Based on our long-term follow-up results from 18 cases, we believe that meticulous preoperative planning for each case is necessary. Intra-operative neuromonitoring, along with an experienced neuromonitoring technologist with clear interpretation of IONM changes, is mandatory when handling such complex cases. Three-dimensional CT and navigation enable the surgeon to achieve the best possible screw trajectory, avoiding injury to the vertebral artery or spinal cord, and providing maximal bony purchase for pedicle screws, which serve as strong anchor points over which reduction manoeuvres are attempted to correct vertical translation and retro-odontoid tilt. By doing so, we were able to get a satisfactory reduction and avoid exploring the atlanto-axial facet joint, which is associated with increased blood loss and duration of surgery, along with other complications. Kosnik-Infinger et al. reported the use of navigation in four paediatric patients for occipito-cervical fixation [28]. However, to the best of our knowledge, there is currently no literature describing the use of intraoperative 3D CT scan and navigation technology in occipito-cervical fusion and its long-term results. 

The results of this study, however, must be interpreted in the context of its limitations. The major limitations are the small sample size and the absence of a comparison group, as all such procedures are currently conducted in the manner described here. Additionally, the institutional bias cannot be eliminated, as this study was conducted in a single centre. The strength of the study is the long-term follow-up of cases.

## Conclusions

Occipito-cervical fusion is the procedure of choice for treating pathologies of the CVJ that lead to instability or myelopathy. Intra-operative navigation and 3D CT scan have enabled accurate screw placement, providing firm anchorage for rigid internal fixation and reduction manoeuvres such as distraction and extension, at the occipito-cervical junction, which are always performed under IONM. Adequate foramen magnum decompression with ultra-sonic bone-scalpel, along with a rigid pedicle screw, rod, and plate construct to provide stability for fusion, can obviate the need for facet joint exploration to put in bone graft or spacers. Additionally, optimal correction of the deformity by gentle reduction manoeuvres, rather than total correction of the deformity, is associated with satisfactory outcomes and fewer complications. Our experience with this technique of navigated screw placement and bone grafting, and optimal reduction has shown improvement in clinical, functional, and radiological outcomes.
